# Hnrnpk maintains chondrocytes survival and function during growth plate development via regulating Hif1α-glycolysis axis

**DOI:** 10.1038/s41419-022-05239-0

**Published:** 2022-09-20

**Authors:** Yuyu Chen, Jinna Wu, Shun Zhang, Wenjie Gao, Zhiheng Liao, Taifeng Zhou, Yongyong Li, Deying Su, Hengyu Liu, Xiaoming Yang, Peiqiang Su, Caixia Xu

**Affiliations:** 1 Department of Spine Surgery, Guangdong Provincial Key Laboratory of Orthopedics and Traumatology, the First Affiliated Hospital of Sun Yat-sen University, Guangzhou, 510080 China; 2 Department of Breast Surgery, Affiliated Cancer Hospital & Institute of Guangzhou Medical University, Guangzhou, 510095 China; 3 Department of Orthopaedics, Sun Yat-sen Memorial Hospital of Sun Yat-sen University, Guangzhou, 510120 China; 4 Precision Medicine Institute, the First Affiliated Hospital of Sun Yat-sen University, Guangzhou, 510080 China; 5 Department of Pathophysiology, School of Basic Medical Sciences, Southern Medical University, Guangzhou, 510515 China; 6 Department of Orthopedics, Renmin Hospital of Wuhan University, Wuhan, 430060 China; 7 Research Center for Translational Medicine, the First Affiliated Hospital of Sun Yat-sen University, Guangzhou, 510080 China

**Keywords:** Apoptosis, Bone development

## Abstract

The harmonious functioning of growth plate chondrocytes is crucial for skeletogenesis. These cells rely on an appropriate intensity of glycolysis to maintain survival and function in an avascular environment, but the underlying mechanism is poorly understood. Here we show that Hnrnpk orchestrates growth plate development by maintaining the appropriate intensity of glycolysis in chondrocytes. Ablating Hnrnpk causes the occurrence of dwarfism, exhibiting damaged survival and premature differentiation of growth plate chondrocytes. Furthermore, Hnrnpk deficiency results in enhanced transdifferentiation of hypertrophic chondrocytes and increased bone mass. In terms of mechanism, Hnrnpk binds to *Hif1a* mRNA and promotes its degradation. Deleting Hnrnpk upregulates the expression of Hif1α, leading to the increased expression of downstream glycolytic enzymes and then exorbitant glycolysis. Our study establishes an essential role of Hnrnpk in orchestrating the survival and differentiation of chondrocytes, regulating the Hif1α-glycolysis axis through a post-transcriptional mechanism during growth plate development.

## Introduction

At the embryonic stage, mesenchymal cells from the lateral plate mesoderm and somite condense and differentiate into chondrocytes, forming the cartilage primordia and then the growth plate [1, 2]. Growth plate chondrocytes experience endochondral ossification and determine the longitudinal bone growth of long bones and vertebrae [3]. During the skeletogenesis, growth plate chondrocytes undergo highly coordinated processes including proliferation, hypertrophy, maturation, and apoptosis, and finally constitute three layers called the resting zone (RZ), proliferating zone (PZ), and hypertrophic zone (HZ) according to their histological characteristics [4]. When the delicate process is disturbed, a number of skeletal deformities may occur [5].

The growth plate is physiological hypoxic tissue because of lacking blood vessels. Thus, the glucose metabolism of chondrocytes prefers glycolysis [6]. Glycolysis is an efficient pattern to produce energy under anoxic or hypoxic conditions, where pyruvic acid transforms into lactate rather than undergoing the tricarboxylic acid cycle [7, 8]. The mechanisms for triggering the glycolysis vary in different tissues, while the growth plate chondrocytes depend on the hypoxia-inducible factor 1 (Hif1) [9, 10], a transcription factor mediating hypoxia adaption via regulating the biological process including angiogenesis, anaerobic metabolism, and proliferation [11]. Hif1 is a heterodimer consisting of Hif1 subunit α (Hif1α) and Hif1 subunit β (Hif1β), and Hif1α is hypoxia sensitive, while Hif1β is constitutively expressed [12]. The conditional ablation of Hif1α results in the failure of the transition from oxidative phosphorylation to glycolysis and further the excessive apoptosis of the chondrocytes [13, 14]. Interestingly, excessive glycolysis also leads to uncoordinated growth plate development, followed by the occurrence of skeletal deformity [15]. Thus, the Hif1α-glycolysis axis plays a pivotal role in maintaining the survival and function of the chondrocytes. Nevertheless, the regulator of and mechanism for maintaining the appropriate intensity of glycolysis during the growth plate development remains unclear.

hnRNPK is a kind of RNA binding protein, but it is capable of regulating gene expression via transcriptional and post-transcriptional aspects [16] and widely participates in RNA metabolism, including splicing, translation, and degradation [17–19]. A de novo loss-of-function mutation in the human gene *HNRNPK* has been reported as related to Au-Kline syndrome (AKS), presenting with remarkable deformities in the limbs and spine [20]. hnRNPK has been proven to regulate the osteogenic differentiation of bone marrow mesenchymal cells [21] and the osteoclastogenesis of bone marrow-derived macrophages [22]. However, the role of hnRNPK in growth plate development is still unknown. In the present study, for the first time, we have demonstrated that Hnrnpk regulated the activity of the Hif-1 signaling pathway, and then maintained the appropriate intensity of glycolysis in the chondrocytes to orchestrate growth plate development.

## Materials and methods

### Mice

**Fig. 1:**
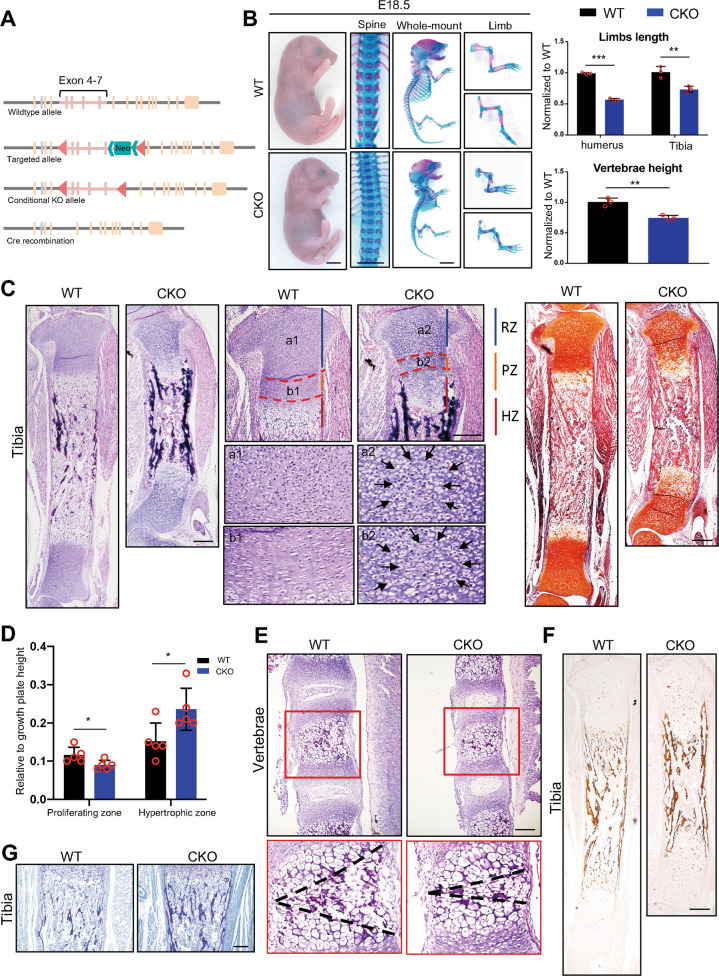
Ablation of Hnrnpk results in the occurrence of dwarfism and abnormal growth plate development. **A** Schematic diagram of the strategy constructing *Hnrnpk*^*loxP*^ mice. **B** General inspection and skeletal preparation of E18.5 WT and CKO embryos (left panel) and quantified comparison of limbs length and vertebrae height between E18.5 WT and CKO embryos (right panel). *n* = 3 biological replicates. Scale bar: 1 mm. **C** H&E staining (left panel) and Safranine O staining (right panel) of tibia of E18.5 WT and CKO embryos. Black arrows: atypical chondrocytes in resting and proliferating zones. Scale bar: 100 μm. **D** Quantification of the height of proliferating zone and hypertrophic zones according to H&E staining of tibia of E18.5 WT and CKO embryos. *n* = 5 biological replicates. **E** H&E staining of lumbar vertebrae of E18.5 WT and CKO embryos. Red boxes: magnification of vertebral growth plate. Black dotted lines: range of ossification center. Scale bar: 100 μm. **F**, **G** von Kassa staining (**F**) and Toluidine blue staining (**G**) of tibia of E18.5 WT and CKO embryos. Scale bar: 100 μm. *p*-value was calculated by two-tailed unpaired Student’s *t*-test. Data were shown as mean ± SD. *p < 0.05; **p < 0.01; ***p < 0.001.

All the mice in this study were bred in the Laboratory Animal Center of Sun Yat-sen University. The *floxed-Hnrnpk* mice were generated by Cyagen Biosciences (Suzhou, China), carrying a pair of *loxP* sites flanking exons 4–7 of the *Hnrnpk* gene (Fig. 1A). The *Col2a1-Cre* and *Col10a1-Cre* mice were the generous gifts from Prof. Xiaochun Bai from Southern Medical University. The *Rosa26-tdTomato* mice were purchased from Jackson Laboratory (JAX 007914). Because sex could not be clearly identified, both male and female embryos were used for analysis. Mice were compared to their littermates so that no specific randomization or blind was used. Genotyping was conducted with the following primers: *Col2a1-Cre* (forward: ATCCGAAAAGAAAACGTTGA; reverse: ATCCAGGTTACGGATATAGT); *Col10a1-Cre* (forward: CTTCCTGCGTTTTGATCATCAGT; reverse: CCACTCACACAACAGTCCTACG); *floxed-Hnrnpk* (forward: GTCTCTCGCTCTGTCTTTGTGGC; reverse: GGAAGGGCTCAGATTAAGTGGCAA); and *Rosa26-tdTomato* (forward1: AAGGGAGCTGCAGTGGAGTA; forward2: CTGTTCCTGTACGGCATGG; reverse1: CCGAAAATCTGTGGGAAGTC; reverse2: GGCATTAAAGCAGCGTATCC). Then, a PCR was conducted according to the following reaction conditions: initial denaturation at 94 °C for 3 min, denaturation at 94 °C for 30 s, annealing at 60 °C for 30 s, extension at 72 °C for 30 s, repeat for 35 cycles, and additional extension at 72 °C for 2 min. We examined three or more littermate groups and showed the representative images.

### Primary chondrocyte cells isolation and cell culture

We isolated the distal femoral and proximal tibial growth plates of the WT or CKO mice at embryonic day (E) 18.5 and cut them into 1-mm^3^ pieces under a stereoscopic microscope (Leica M205FA, Germany). We digested the growth plate tissue with 0.25% trypsin for 10 min at 37 °C. Then, the tissue was digested with 0.2% collagenase type II (Life Technologies, USA) and shaken in an incubated air shaker (Thermo MaxQ4000, USA) at a speed of 250 rpm at 37 °C for 12 h. Chondrocytes were seeded in a 6-well plate and cultured at 37 °C with 5% CO_2_. DMEM/F12 with L-glutamine (Gibco, USA) supplemented with 1% penicillin–streptomycin solution (Gibco, USA) and 10% fetal bovine serum (PAN, Germany) was used as the culture medium. Chondrocytes were used within three passages for the hypoxia studies. When grown to ~75% confluence, the chondrocytes were exposed to normoxic (20% O_2_) or hypoxic (1% O_2_) conditions for 48 h before adding Chetomin (GLPBIO, USA, GC17405, 25 nM) or 3-BrPA (MCE, USA, HY19992, 2 μM).

### qPCR

Total RNA was extracted from cartilage tissue and cultured cells using TRIZOL reagent (Invitrogen, USA), and cDNA was synthesized using 1000 ng of total RNA with a Prime-Script RT reagent kit (TaKaRa, Japan) according to the manufacturer’s protocol. A qPCR was performed to amplify the cDNA on a Light Cycler 480 Real-Time PCR system (Roche Light Cycler 480, Switzerland) using TB Green Premix Taq II (TaKaRa, Japan) and the corresponding primers. The sequences of the primers were listed in Supplementary Table 1. The 2^−ΔΔCt^ method was used to calculate the relative expression levels, and *Actb* served as the internal control for normalization.

### Immunoblotting analysis

We performed western blotting (WB) according to standard procedures. The following primary antibodies were used: anti-Hnrnpk (Abcam 39975, 1:5000), anti-Bax (Cell Signaling Technology 14796, 1:1000), anti-p21 (Abcam 109520, 1:1000), anti-Ihh (Abcam 52919, 1:1000), anti-Runx2 (Abcam 192256, 1:1000), anti-Mmp13 (Abcam 219620, 1:500), anti-Sox9 (Abcam 185966, 1:1000), anti-Hif1α (Cell Signaling Technology 36169, 1:250), anti-Gapdh (Proteintech 60004-1, 1:2000), anti-Pfkfb3 (Abcam 181861, 1:500), anti-Ldha (Proteintech 19987, 1:2000), and anti-β-Actin (Affinity AF7018, 1:2000). The following secondary antibodies were used: goat anti-rabbit IgG H&L (Abcam ab205718, 1:2000) and goat anti-mouse IgG H&L (Abcam ab205719, 1:2000). The Densitometry analysis of WB was exerted using software Image J (v 1.51). The original western blots were shown in the Supplementary material Western blots.

### Skeletal preparation

We performed the skeletal preparation as previously described [23] and photographed the images under a stereoscopic microscope (Zeiss Axio Zoom v16, Germany) in glycerol.

### Histological analysis

The embryos were eviscerated, and the limbs were dissected and fixed in 4% paraformaldehyde for 48 h at 4 °C. All the limbs were embedded in paraffin or OCT. Then the embedded limbs were processed for paraffin sectioning at a thickness of 5 μm or frozen sectioning at a thickness of 6 μm. The paraffin sections were dewaxed in xylene and rehydrated in a graded concentration of alcohol before staining. For von Kossa staining, the undecalcified sections were stained with 1% silver nitrate solution under a 100-W light bulb for 1 h. After being rinsed in three changes of distilled water, the sections were incubated with 5% sodium thiosulfate for 5 min. After washing with distilled water, the nuclei were counterstained with 0.1% nuclear fast red solution (Solarbio, China) for 5 min. For Toluidine blue staining, the sections were stained with 0.5% Toluidine blue staining solution for 20 min. For tartrate-resistant acid phosphatase (TRAP) staining, the TRAP activity was detected in the paraffin sections using a TRAP staining kit (SLBT1113, Sigma, USA) according to the manufacturer’s instructions. All slides were dehydrated through graded alcohol and cleared in xylene. Coverslips were placed with neutral resins (Servicebio, China). The slides were photographed using microscopy (Olympus BX63, Japan).

### 5-Bromo-2’-deoxyuridine (BrdU) incorporation assay

The pregnant mice were given BrdU (MCE, HY-15910, USA, 100 mg/kg) through intraperitoneal injection 2 h before sacrifice. The sections were stained with the anti-BrdU antibody (Abcam 6326, 1:200) according to the procedure of immunofluorescent staining.

### Immunofluorescent staining

After being sectioned at a thickness of 6 μm and mounted onto slides, the frozen sections from the E13.5, E15.5, and E18.5 forelimbs were air-dried and washed three times with PBS. Sections were incubated in PBS containing 0.1% Triton X-100 for 5 min at room temperature. After being blocked with 5% BSA in PBS at room temperature for 30 min, the sections were incubated with the following primary antibodies overnight at 4 °C: anti-Hnrnpk (Abcam ab52600, 1:250), anti-CD31 (Cell Signaling Technology 3528, 1:1000), anti-Hif1α (Bioss bs-0737R, 1:200), anti-Ctsk (Abcam 187647, 1:500), anti-Sp7 (Abcam 209484), anti-Sox9 (Abcam 185966, 1:1000), anti-Col10a1 (Abcam 49945, 1:1000), anti-Mmp13 (Abcam 219620, 1:250), and anti-Runx2 (Abcam 192256, 1:500). The sections were washed three times with PBS, followed by species-matched Alexa Fluor 555 or Alexa Fluor 488 for 1 h. The nuclei were counterstained with DAPI (Cell Signaling Technology, USA) for 5 min. Slides were washed with PBS, and then the antifade mounting medium (Servicebio, China) was placed between the coverslips and slides. The slides were photographed with fluorescent microscopy (Olympus BX63, Japan). The quantification was exerted using software Image J (v 1.51).

### TdT-mediated dUTP nick end labeling (TUNEL) assay

The paraffin sections from the E18.5 limbs were deparaffinized in xylene, rehydrated through graded alcohols, and washed with distilled water. We then exerted TUNEL staining according to the manufacturer’s instructions for the one-step TUNEL Apoptosis Assay Kit (Beyotime, China). Briefly, after incubation with 20 μg/ml proteinase K for 30 min at 37 °C, the sections were washed three times with PBS. The TUNEL detective mixture was pipetted onto the sections, followed by incubation at 37 °C for 60 min in darkness. Then, the sections were washed three times with PBS. The slides were mounted with an antifade mounting medium (Servicebio, China) under the coverslips and photographed using fluorescent microscopy (Olympus, BX63, Japan).

### Bone CT scanning and BMD measurements

The limbs of the one-month/two-month WT and cCKO mice were dissected and fixed in 4% paraformaldehyde for 48 h at 4 °C; they were then stored in 70% ethanol for micro-CT scanning (SCANCO Medical AG, Switzerland). Quantitative volumetric measurements of trabecular micro-structures were conducted on the distal femoral metaphysis region of 50 micro-tomographic slices (500 μm below the growth plate), focusing on the primary spongiosa. We measured the bone micro-structure parameters using the Siemens Preclinical Imaging System, including bone mineral density (BMD), bone volume/tissue volume ratio (BV/TV), trabecular thickness (Tb.Th), trabecular number (Tb.N), and trabecular separation (Tb.Sp).

### RNA-seq

Total RNA was extracted from the cartilage growth plate of the E18.5 WT and CKO embryos and the libraries were constructed using the VAHTS Stranded mRNA-seq Library Prep Kit for Illumina v2. We checked the library’s quality using Qubit 2100, and the concentrations were determined from the analysis profiles. Genes expressed with a log_2_(fold change) >1 or <−1, *p-*value < 0.05 were identified as differentially expressed genes, and we performed the GO enrichment analysis and KEGG pathway analysis using DAVID [24].

### Adenovirus infection

When chondrocytes had grown to ~75% confluence in a 6-well plate, the medium was changed to 1 ml of DMEM/F12. The *Ad-GFP* and *Ad-Cre* were added to the medium with a concentration of 10^8^ PFU/ml and cultured under standard conditions. Two hours later, 1 ml of DMEM/F12 with L-glutamine supplemented with 1% penicillin–streptomycin solution and 10% fetal bovine serum was added into each well. After 12–16 h, the medium was changed. After 24 h, the fluorescent signal was observed under a fluorescent microscope (Leica DMi8). Before the next step of the experiment, chondrocytes were cultured for another 24 h.

### Measurement of mRNA half-life

After adenovirus infection, the chondrocytes were treated with 10 μg/ml actinomycin D (MCE HY-17559) for 0, 0.5, 1, 2, 4, and 6 h. Then, the RNA was extracted, and the equal RNA was used for synthesizing cDNA and exerted RT-qPCR to determine the level of mRNA. Half-life was calculated using the following formula: T_1/2_ = 0.3t/log(D1/ D2) [25, 26].

### Glycolysis measurement

The measurement of glucose concentration was performed according to the Glucose (HK) Assay Kit (Sigma, USA) protocol. 50 µl of the cell culture supernatant was incubated with 1 ml of the assay reagent for 15 min at room temperature, and the absorbance at 340 nm versus deionized water was measured (Victor Nivo 5S, Finland). The measurement of lactate concentration was performed according to the L-lactate assay kit I (Eton Biosciences, USA) protocol. 50 µl of the cell culture supernatant was incubated with 50 µl of L-lactate assay solution and incubated for 30 min at 37 °C. The reaction was stopped by adding 50 μl of 0.5 M acetic acid per well followed by brief gentle agitation. The absorbance at 490 nm was measured using a microplate reader (Victor Nivo 5S, Finland).

### RNA-IP (RIP)/RIP-PCR/RIP-qPCR

RIP was performed according to the RIP-Assay Kit (MBL, Japan) protocol. Briefly, 1 × 10^7^ cells per sample were detached from the culture dish by scraping and then collected. The Protein G magnetic beads (Thermo Scientific 88847) were immobilized with primary antibodies including anti-Hnrnpk (Bethyl A300-676A-1) and anti-rabbit IgG (Abcam 6702). After lysis, 10 μl of lysate was saved as input, and the residue cell lysate supernatant was transferred to the tube containing antibody-immobilized Protein G magnetic beads, followed by incubation of the tube with rotation for 3 h at 4 °C. After the fourth wash of the bead-RNP complex, 100 μl of the mixture was saved to check the quality by WB, and the residue mixture was used for extracting RNA. Then, the cDNA was synthesized with a Prime-Script RT reagent kit (TaKaRa) according to the manufacturer’s protocol. A PCR was performed to detect the binding between Hnrnpk and mRNA, and the qPCR was performed for quantified comparison. The 2^−ΔΔCt^ method was used to calculate the relative binding levels, and the corresponding input served as the control for normalization.

### Statistical analysis

Quantification was done in at least three independent experimental groups. The analysis of all statistics was carried out using SPSS v13. A two-tailed Student’s *t*-test was used between two groups to determine the significance, while a one-way ANOVA with Tukey’s post hoc test was used to compare differences between multiple groups. In all cases, a *p*-value < 0.05 was considered significant, and the results were presented as mean ± standard deviation (SD).

## Results

### Hnrnpk is required for normal growth plate development and its ablation in chondrocytes leads to dwarfism

First, we detected the expression of Hnrnpk in developing long bone during the process of embryonic growth plate development using consecutive humerus sections. At E13.5, Hnrnpk was mainly expressed in cartilage primordia coincident with the expression of SRY-box transcription factor 9 (Sox9), and some scattered signals were also detected in adjacent soft tissue (Supplementary Fig. S1A). At E15.5, when the primary ossification center (POC) was formatted, Hnrnpk was widely expressed in the long bone, ossification center, and adjacent soft tissue (Supplementary Fig. S1B). At E18.5, we detected a similar pattern as at E15.5, while Hnrnpk was partially expressed in proliferating chondrocytes, different from the entire expression in the resting and hypertrophic zones (Supplementary Fig. S1C). Taken together, Hnrnpk was widely expressed in developing long bones, suggesting it might possess a regulatory function in bone growth.

To explore the function of Hnrnpk in bone growth, we constructed the *floxed-Hnrnpk* by inserting two *loxP* sites composing exons 4–7 and ablated Hnrnpk in chondrocytes via mating *Hnrnpk*^*fl/fl*^ with *Col2a1-Cre* mice [27] (Fig. 1A, Supplementary Fig. S1D). Using protein extracted from the growth plate cartilage of E15.5 *Col2a1-Cre;Hnrnpk*^*fl/fl*^ (conditional knockout, CKO) mice, a WB confirmed the effective knockout of Hnrnpk compared with *Hnrnpk*^*fl/fl*^ (wild type, WT) (Supplementary Fig. S1E). Immunostaining using anti-Hnrnpk with the E15.5 CKO humerus also confirmed the specific ablation of Hnrnpk in the growth plate (Supplementary Fig. S1F). The CKO mice died soon after delivery because of suffocation. We sacrificed the pregnant mice at E18.5 and observed that the CKO embryos exhibited severe dwarfism, manifesting shortened limbs and spine, compressed vertebrae, and the constriction of the thoracic cavity, which resulted in the suffocation (Fig. 1B). Whole-mount skeletal preparation showed the significantly shortened length of the long bone and decreased height of the vertebrae (Fig. 1B). However, there was no significant difference between the WT and CKO embryos according to appearance and skeletal preparation at E15.5 (Supplementary Fig. S2A). Therefore, the dwarfism caused by ablating Hnrnpk indicated that Hnrnpk was required during bone growth.

To depict the changes in the Hnrnpk null growth plate, we isolated the tibia of E18.5 WT and CKO embryos and observed the structure of the growth plate. The histological characteristics of the CKO embryos exhibited shortened growth plate, hypocellularity, and disordered arrangement of the three layers (Fig. 1C). Specifically, there were some atypical chondrocytes existing in the center of the resting zone and proliferating zone (Fig. 1C, black arrow). The atypical chondrocytes were eccentrically swollen, composed of a significantly increased cytoplasm to nucleus ratio. In the growth plate of CKO embryos, the distribution of hypertrophic cells became sparse, and the range of hypertrophic zone became wider while the range of the proliferating zone became narrower (Fig. 1C, D). Similar changes were also observed in the CKO vertebrae with a delayed formation of the ossification center (Fig. 1E). Significantly increased bone mass in the CKO embryos was detected using von Kassa staining (Fig. 1F) and the Toluidine blue staining (Fig. 1G). However, there was no significant histological change detected, except the delayed formation of POC in the E15.5 CKO embryos (Supplementary Fig. S2B, black arrow). Overall, the Hnrnpk null growth plate exhibited a disordered arrangement, manifesting in a decreased range of resting and proliferating zones, crammed with atypical chondrocytes, increased range of hypertrophic zone, and osteosclerosis.

### Ablation of Hnrnpk results in the damaged survival and premature differentiation of growth plate chondrocytes

**Fig. 2:**
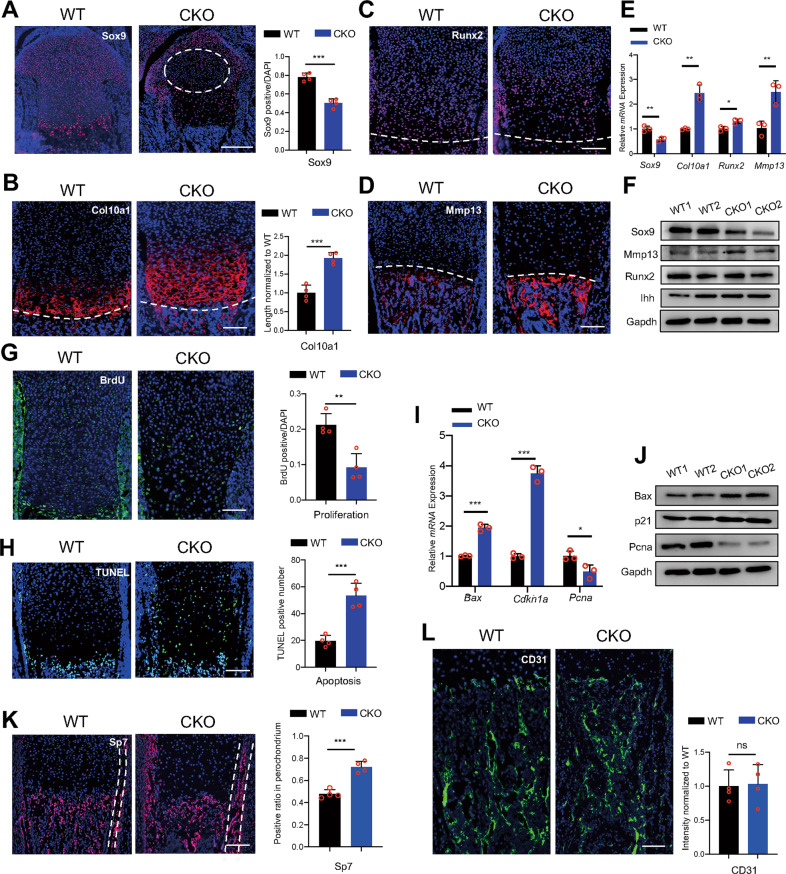
Loss of Hnrnpk results in damaged survival and premature differentiation of growth plate chondrocytes. **A** Immunostaining of Sox9 of tibia of E18.5 WT and CKO embryos (left panel) and the quantification of Sox9 positive cells (right panel). Dotted circle: region contained atypical chondrocytes. *n* = 4 biological replicates. Scale bar: 100 μm. **B** Immunostaining of Col10a1 of tibia of E18.5 WT and CKO embryos (left panel) and measurement of Col10a1 positive range (right panel). Dotted lines: boundary between hypertrophic zone and POC. *n* = 4 biological replicates. Scale bar: 100 μm. **C**, **D** Immunostaining of Runx2 (**C**) and Mmp13 (**D**) of tibia of E18.5 WT and CKO embryos. Dotted lines: boundary between hypertrophic zone and POC. Scale bar: 100 μm. **E** mRNA expression level of *Sox9*, *Col10a1*, *Mmp13*, and *Runx2* of E18.5 WT and CKO growth plate cartilage. *n* = 3 biological replicates. **F** Protein level of Sox9, Mmp13, Runx2, and Ihh of E18.5 WT and CKO growth plate cartilage. **G**, **H** BrdU assay (**G**) and TUNEL assay (**H**) of tibia of E18.5 WT and CKO embryos (left panel) and the quantification of BrdU and TUNEL positive cells (right panel). *n* = 4 biological replicates. Scale bar: 100 μm. **I** mRNA expression level of *Bax*, *Cdkn1a*, and *Pcna* of E18.5 WT and CKO growth plate cartilage. *n* = 3 biological replicates. **J** Protein level of Bax, p21, and Pcna of E18.5 WT and CKO growth plate cartilage. **K**, **L** Immunostaining of Sp7 (**K**) and CD31 (**L**) in POC of tibia from E18.5 WT and CKO embryos (left panel) and the quantification of Sp7 and CD31 staining (right panel). Dotted lines: perichondrium. *n* = 4 biological replicates. Scale bar: 100 μm. *p*-value was calculated by two-tailed unpaired Student’s *t*-test. Data were shown as mean ± SD. *p < 0.05; **p < 0.01; ***p < 0.001; ns: not significant.

Growth plate chondrocytes experience proliferation and differentiation during development, and their disorder results in the malfunction of the growth plate. Sox9 was originally expressed in the normal resting and proliferating zones, but the ratio of Sox9-positive cells decreased in the E18.5 CKO embryos (Fig. 2A). Interestingly, the atypical chondrocytes manifested Sox9-negative (Fig. 2A, white round). Coincident with the increase of the hypertrophic zone, the Collagen X, type 1 (Col10a1) positive range expanded longitudinally significantly in the E18.5 CKO embryos (Fig. 2B). The marker of early-hypertrophic chondrocytes Runx family transcription factor 2 (Runx2) showed no significant difference between the WT and CKO embryos (Fig. 2C), while the indicator of late-hypertrophic chondrocytes Matrix metallopeptidase 13 (Mmp13) increased significantly in the E18.5 CKO embryos (Fig. 2D), indicating the premature differentiation of chondrocytes. qPCR and WB were exerted using RNA and protein extracted from the E18.5 growth plate cartilage, and the results showed decreased expression of Sox9 and increased expression of Mmp13, Col10a1, and Indian hedgehog (Ihh) in the CKO embryos (Fig. 2E, F), similar to immunostaining. Then, the BrdU assay and TUNEL assay were exerted, and the depressed proliferation (Fig. 2G) and excessive apoptosis were detected (Fig. 2H) in the E18.5 CKO embryos. The upregulation of p21 and BCL2 associated X (Bax) and downregulation of Proliferating cell nuclear antigen (Pcna) in the E18.5 CKO growth plate cartilage also indicated the restriction of the cell cycle and increased apoptosis (Fig. 2I, J). Concordant with the increased osteogenesis, the expression of early osteoblast marker Sp7 was markedly increased in CKO bone marrow and perichondrium, which also expressed Col2a1, in the E18.5 CKO embryos (Fig. 2K). However, the indicator of the vessel endothelial cells CD31 showed no significant difference between the WT and CKO embryos (Fig. 2L). At E15.5, the above indicators showed no difference except the decreased signal of Mmp13 in the CKO embryos, suggesting the delayed formation of POC (Supplementary Fig. S2C–F). Taken together, our data showed that ablating Hnrnpk resulted in the damaged survival and premature differentiation of chondrocytes.

### Osteosclerosis in CKO mice results from the enhanced transdifferentiation of hypertrophic chondrocytes

**Fig. 3:**
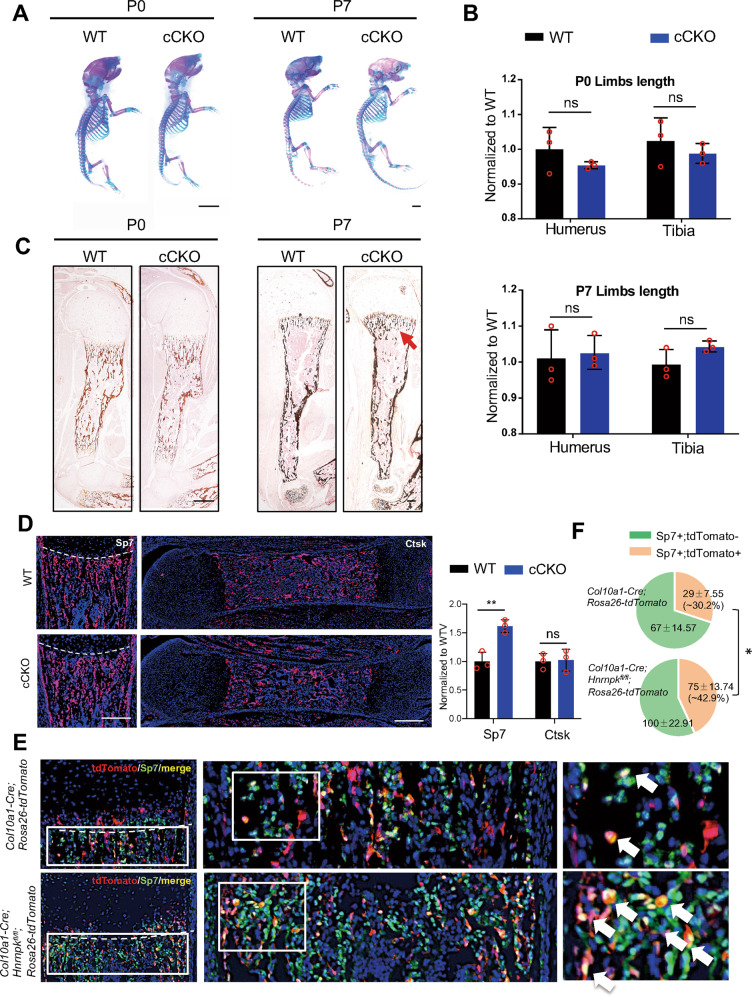
Ablating Hnrnpk in hypertrophic chondrocytes results in enhanced transdifferentiation potential. **A** Skeletal preparation of P0 (left panel) and P7 (right panel) WT and cCKO mice. Scale bar: 1 mm. **B** Quantification of limb length of P0 (top) and P7 (bottom) WT and cCKO mice. *n* = 3 biological replicates. **C** von Kassa staining of humerus of P0 and P7 WT and cCKO mice. Red arrow: excessive mineral bone in cCKO mice. Scale bar: 100 μm. **D** Immunostaining of Sp7 (left panel) and Cathepsin K (Ctsk) (right panel) of femur of P0 WT and cCKO mice (left panel) and the quantification of Sp7 and Ctsk positive cells (right panel). Dotted lines: boundary between hypertrophic zone and POC. *n* = 3 biological replicates. Scale bar: 100 μm. **E** Immunostaining of Sp7 of P0 *Col10a1-Cre;Rosa26-tdTomato* and *Col10a1-Cre;Hnrnpk*^*fl/fl*^*;Rosa26-tdTomato*. White boxes: magnification of metaphysis. White arrow: tdTomato and Sp7 double-positive cells. Scale bar: 100 μm. **F** Total quantity and ratio of single positive cells and double-positive cells in (**E**) of P0 WT and cCKO mice. Statistical analysis was exerted upon a ratio of double-positive cells between WT and cCKO mice. *n* = 3 biological replicates. *p*-value was calculated by two-tailed unpaired Student’s *t*-test. Data were shown as mean ± SD. *p < 0.05; **p < 0.01; ns: not significant.

A previous study had proved that some of the late-hypertrophic chondrocytes died while the others transdifferentiated into osteoblasts in the bone marrow [28]. To study whether the osteosclerosis in the E18.5 CKO mice resulted from enhanced transdifferentiation of hypertrophic chondrocytes, we knocked out Hnrnpk in the hypertrophic chondrocytes using *Col10a1-Cre* [29, 30]. The *Col10a1-Cre;Hnrnpk*^*fl/fl*^ (cCKO) mice were born normally in Mendelian law. There was no significant abnormality, including the limb length and height, between the WT and cCKO mice at P0 or P7 (Fig. 3A, B, Supplementary Fig. S3A). The H&E staining showed no difference between the WT and cCKO mice at E16.5, P0, and P7, except the slightly increased range of the hypertrophic zone at P7 cCKO mice (Supplementary Fig. S3B). However, increased bone mass was detected in the P7 cCKO mice (Fig. 3C, red arrow). Though there was no significant change in bone mass between the P0 WT and cCKO mice, the expression of Sp7 increased significantly in the metaphysis, without change in the number or function of osteoclasts in the P0 cCKO mice (Fig. 3D, Supplementary Fig. S3C). To illustrate whether the increased quantity of osteoblasts was due to the enhanced transdifferentiation of hypertrophic chondrocytes lacking Hnrnpk, we traced the hypertrophic chondrocytes by mating cCKO mice with *Rosa26-tdTomato* mice. The number of Sp7-positive cells increased (Fig. 3E), as well as the number and ratio of Sp7-tdTomato double-positive cells in the P0 *Col10a1-Cre;Hnrnpk*^*fl/fl*^*;Rosa26-tdTomato* mice versus the *Col10a1-Cre;Rosa26-tdTomato* mice (~42.9% vs ~30.2%) (Fig. 3F). The 1- and 2-month-old cCKO mice exhibited slightly increased bone mass though not statistically different (Supplementary Fig. S3D), which might be due to the fact that a considerable portion of osteoblasts originated from the periosteum and bone marrow mesenchymal cells rather than the growth plate in the juvenile stage [31, 32]. Thus, our data indicated that knocking out Hnrnpk in hypertrophic chondrocytes resulted in enhanced transdifferentiation and then increased bone mass.

### Hnrnpk deficiency elevates the activity of Hif-1 signaling pathway via regulating *Hif1a* mRNA

**Fig. 4:**
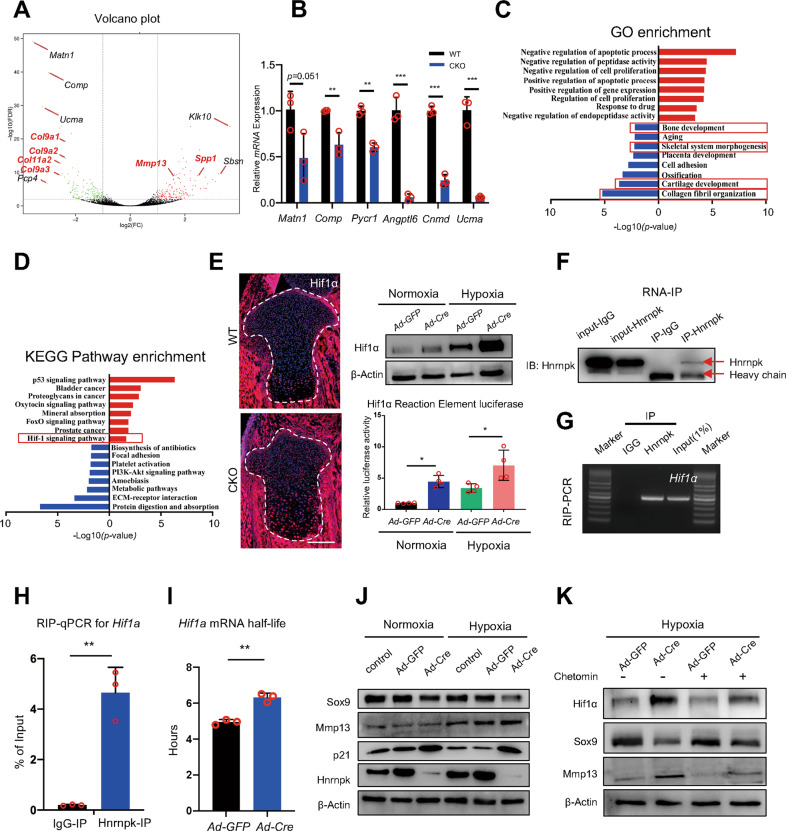
Elevated activity of Hif-1 signaling pathway in CKO growth plate chondrocytes. **A** Volcano plot of RNA-seq. **B** mRNA expression level of significantly downregulated genes (*Matn1*, *Comp*, *Pycr1*, *Angptl6*, *Cnmd*, and *Ucma*) in E18.5 CKO growth plate cartilage compared to WT. *n* = 3 biological replicates. **C**, **D** GO enrichment analysis (**C**) and KEGG pathway enrichment analysis (**D**) of up- and down-regulated genes (Log_2_FC > 1 or < -1, *p-*value < 0.05) according to RNA-seq results. Red boxes: items we focus on. **E** The expression of Hif1α of tibia of E18.5 WT and CKO embryos (left panel), and protein level of Hif1α (right panel, top) and the luciferase activity (right panel, bottom) of *Hnrnpk*^*fl/fl*^ chondrocytes infected with *Ad-GFP* or *Ad-Cre* under normoxic or hypoxic conditions. *n* = 4 biological replicates. Scale bar: 100 μm. **F** Hnrnpk protein level of supernatant immunoprecipitated with antibodies of Hnrnpk or IgG. **G**, **H** RIP-PCR (**G**) and RIP-qPCR (**H**) were used to determine the level of binding between Hnrnpk and *Hif1a* mRNA. IgG IP was used as a specificity control. *n* = 3 biological replicates. **I** Half-life of *Hif1a* mRNA of *Hnrnpk*^*fl/fl*^ chondrocytes infected with *Ad-GFP* or *Ad-Cre*. *n* = 3 biological replicates. **J** Protein level of Sox9, Mmp13, p21, and Hnrnpk of *Hnrnpk*^*fl/fl*^ chondrocytes infected with *Ad-GFP* or *Ad-Cre* under normoxic or hypoxic conditions. **K** Protein level of Hif1α, Sox9, and Mmp13 in *Hnrnpk*^*fl/fl*^ chondrocytes treated with DMSO or Chetomin after being infected with *Ad-GFP* or *Ad-Cre* under hypoxic condition. *p*-value was calculated by one-way ANOVA followed by Tukey’s multiple comparisons tests (**E**) or two-tailed unpaired Student’s *t*-test (**B**, **H**, **I**). Data were shown as mean ± SD. *p < 0.05; **p < 0.01; ***p < 0.001.

To reveal the mechanism of the functional change of the Hnrnpk null growth plate, we isolated the growth plate cartilage of the E18.5 WT and CKO embryos and exerted RNA-sequencing (RNA-seq). The genes upregulated >2 folds and downregulated >2 folds whose *p-*value < 0.05 were deemed to be significantly differentially expressed. Compared to the WT groups, the genes relative to the cartilage matrix including *Matrilin1* (*Matn1*), *Cartilage oligomeric matrix protein* (*Comp*), and *Upper zone of growth plate and cartilage matrix associated* (*Ucma*) were downregulated most significantly in the CKO groups (Fig. 4A). A qPCR was exerted using the E18.5 growth plate cartilage to confirm the changes in the Hnrnpk null growth plate (Fig. 4B). However, the genes relative to osteogenesis including *Secreted phosphoprotein 1* (*Spp1*), *Mmp13* were significantly upregulated (Fig. 4A). Then, the differentially expressed genes were subjected to Gene Ontology (GO) enrichment analysis, and the downregulated genes in CKO groups were enriched for items including collagen fibril organization, cartilage development, and bone development (Fig. 4C). The upregulated genes in the CKO groups were subjected to Kyoto Encyclopedia of Genes and Genomes (KEGG) pathway enrichment analysis and the result showed that the Hif-1 signaling pathway was significantly enriched (Fig. 4D). The Hif-1 signaling pathway mainly depended on the transcription factor Hif1, and its subunit Hif1α was hypoxia sensitive and essential for growth plate development [33]. The upregulation of Hif1α in the E18.5 CKO embryos was detected (Fig. 4E), as well as in the *Hnrnpk*^*fl/fl*^ chondrocytes infected with *Ad-Cre*, especially under hypoxic condition (1% oxygen) (Fig. 4E). Concordant with the increased expression of Hif1α, the luciferase activity of the Hif1 reaction element (HRE) [34] also elevated significantly in *Hnrnpk*^*fl/fl*^ chondrocytes infected with *Ad-Cre*, especially under hypoxic condition (Fig. 4E). Thus, the elevated Hif-1 signaling pathway activity in the Hnrnpk null chondrocytes was due to the increased expression of Hif1α.

Hnrnpk possessed the capacity to bind RNA and regulates its stability [16]. To explore whether Hnrnpk regulated the stability of *Hif1a* mRNA, we exerted RNA immunoprecipitation (RNA-IP, RIP) using primary chondrocytes and confirmed the efficiency of immunoprecipitation (Fig. 4F). Then, the RIP-PCR and RIP-qPCR confirmed a significant binding between Hnrnpk and *Hif1a* mRNA (Fig. 4G, H). To test the stability of *Hif1a* mRNA in Hnrnpk null chondrocytes, the chondrocytes were treated with actinomycin D to determine the half-life, and the loss of Hnrnpk significantly increased the half-life of *Hif1a* mRNA (Fig. 4I). These results suggested that Hnrnpk bound to the transcript of *Hif1a* and promoted its degradation, and the loss of Hnrnpk resulted in the elevated activity of the Hif-1 signaling pathway.

To determine whether the increased Hif-1 signaling pathway activity resulted in the functional change of Hnrnpk null chondrocytes, *Hnrnpk*^*fl/fl*^ chondrocytes were infected with *Ad-Cre* and cultured under hypoxic condition to activate the Hif-1 signaling pathway. No significant difference of Sox9 and Mmp13 under normoxic condition (20% oxygen) was detected between the *Hnrnpk*^*fl/fl*^ chondrocytes infected with *Ad-GFP* and *Ad-Cre* (Fig. 4J). However, under hypoxic condition, the decreased expression of Sox9 and increased expression of Mmp13 were detected in the Hnrnpk ablation chondrocytes (Fig. 4J). Then, the *Hnrnpk*^*fl/fl*^ chondrocytes were treated with Chetomin [35], Hif1α inhibitor, after being infected with *Ad-GFP* or *Ad-Cre* under hypoxic condition, and the decrease of Sox9 and increase of Mmp13 were significantly reversed in the Hnrnpk ablation chondrocytes (Fig. 4K). Therefore, the functional change of the chondrocytes resulted from the elevated activity of Hif-1 signaling pathway.

### Elevated activity of Hif-1 signaling pathway in Hnrnpk null chondrocytes increases the intensity of glycolysis

**Fig. 5:**
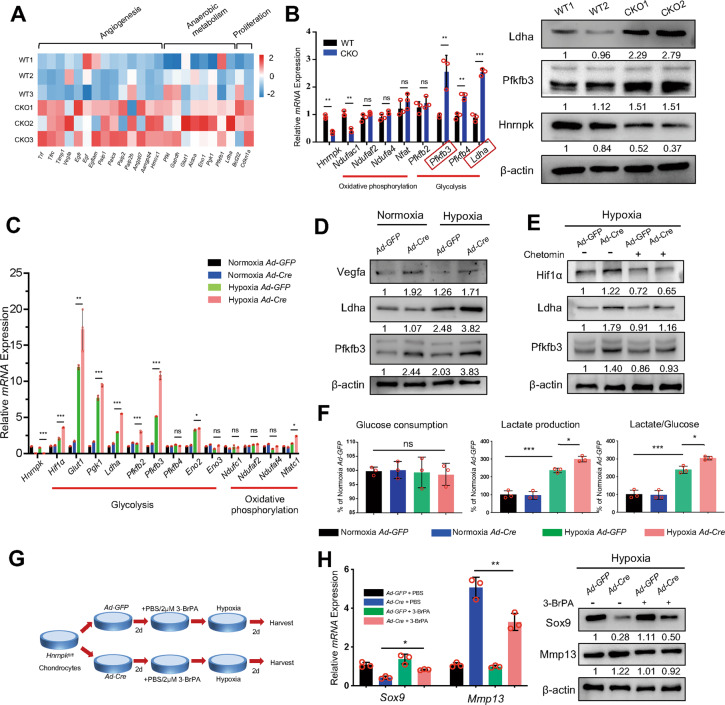
Elevated activity of Hif-1 signaling pathway in Hnrnpk null chondrocytes increases glycolytic intensity. **A** mRNA fold changes of target genes of Hif-1 signaling pathway of E18.5 CKO growth plate cartilage compared to WT according to RNA-seq results. **B** mRNA expression level of key enzymes of oxidative phosphorylation and glycolysis of E18.5 WT and CKO growth plate cartilage (left panel). Protein level of Pfkfb3 and Ldha of E18.5 WT and CKO growth plate cartilage (right panel). Red boxes: most active glycolytic enzymes in chondrocytes. *n* = 3 biological replicates. Densitometry results were expressed as fold change in protein levels compared with WT1 after normalized to β-actin. **C** mRNA expression level of key enzymes of oxidative phosphorylation and glycolysis of *Hnrnpk*^*fl/fl*^ chondrocytes infected with *Ad-GFP* or *Ad-Cre* under normoxic or hypoxic conditions. Statistical analysis was exerted between Hypoxia *Ad-GFP* and Hypoxia *Ad-Cre*. *n* = 3 biological replicates. **D** Protein level of Vegfa, Ldha, and Pfkfb3 of *Hnrnpk*^*fl/fl*^ chondrocytes infected with *Ad-GFP* or *Ad-Cre* under normoxic or hypoxic conditions. Densitometry results were expressed as fold change in protein levels compared with chondrocytes infected with *Ad-GFP* under normoxic condition after normalized to β-actin. **E** Protein level of Hif1α, Ldha, and Pfkfb3 of *Hnrnpk*^*fl/fl*^ chondrocytes treated with DMSO or Chetomin after being infected with *Ad-GFP* or *Ad-Cre* under hypoxic condition. Densitometry results were expressed as fold change in protein levels compared with chondrocytes infected with *Ad-GFP* under hypoxic condition after normalized to β-actin. **F** Glucose consumption, lactate production, and ratio of lactate/glucose of *Hnrnpk*^*fl/fl*^ chondrocytes infected with *Ad-GFP* or *Ad-Cre* under normoxia or hypoxic conditions. *n* = 3 biological replicates. **G** Diagram indicated the strategy of treating *Hnrnpk*^*fl/fl*^ chondrocytes with PBS or 3-BrPA after being infected with *Ad-GFP* or *Ad-Cre* under hypoxic condition. **H** mRNA expression level and protein level of Sox9 and Mmp13 in *Hnrnpk*^*fl/fl*^ chondrocytes treated with PBS or 3-BrPA after being infected with *Ad-GFP* or *Ad-Cre* under hypoxic condition. *n* = 3 biological replicates. Densitometry results were expressed as fold change in protein levels compared with chondrocytes infected with *Ad-GFP* under hypoxic condition after normalized to β-actin. *p*-value was calculated by one-way ANOVA followed by Tukey’s multiple comparisons tests (**C**, **F**, **H**) or two-tailed unpaired Student’s *t*-test (**B**). Data were shown as mean ± SD. *p < 0.05; **p < 0.01; ***p < 0.001; ns: not significant.

In growth plate development, the Hif-1 signaling pathway mainly influences angiogenesis, anaerobic metabolism, and cell proliferation [9]. Furthermore, the pattern and intensity of the glucose metabolism were more important for Hif1α to regulate the growth plate [14]. We first detected the increased expression of genes involving anaerobic metabolism in CKO groups according to the RNA-seq results (Fig. 5A). We also confirmed the increase of intensity of glycolysis in the E18.5 CKO embryos, presented by the increased expression of 6-phosphofructo-2-kinase/fructose-2,6-biphosphatase 3 (Pfkfb3) and Lactate dehydrogenase A (Ldha) (Fig. 5B), which were more active in the chondrocytes [36, 37]. To confirm that the elevated level of glycolysis was due to the increased Hif1α expression rather than decreased oxygen level in vivo, we treated the *Hnrnpk*^*fl/fl*^ chondrocytes with Chetomin after being infected with *Ad-GFP* or *Ad-Cre* under hypoxic condition. The expression of glycolytic enzymes increased in the Hnrnpk ablation chondrocytes (Fig. 5C, D) but recovered after being treated with Chetomin (Fig. 5E). Then, due to the elevated expression of glycolytic enzymes, the increased production of lactate and the ratio between lactate and glucose were detected in the Hnrnpk ablation chondrocytes under hypoxic condition (Fig. 5F). Therefore, these results indicated that ablating Hnrnpk in chondrocytes increased the expression of Hif1α, and elevated Hif1α increased the expression of its downstream glycolytic enzyme and then the excessive glycolysis.

To test whether the functional change of Hnrnpk null chondrocytes resulted from the excessive intensity of glycolysis, the *Hnrnpk*^*fl/fl*^ chondrocytes were treated with 3-Bromopyruvic acid (3-BrPA) [38], the hexokinase-II inhibitor, to suppress glycolysis after being infected with *Ad-GFP* or *Ad-Cre* under hypoxic condition (Fig. 5G). After the treatment of 3-BrPA, the decrease of Sox9 and increase of Mmp13 could be rescued in the Hnrnpk ablation chondrocytes (Fig. 5H). Therefore, the results indicated that the loss of Hnrnpk led to the excessive intensity of glycolysis and further the functional change of the chondrocytes.

### Suppressing the intensity of glycolysis in CKO embryos partially rescues the dwarfism

**Fig. 6:**
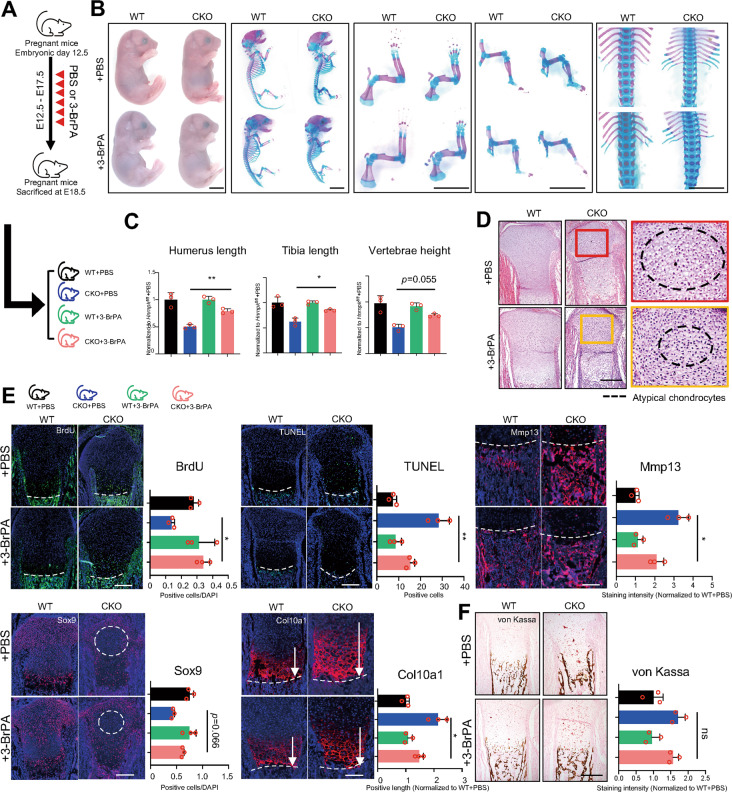
Suppressing glycolytic intensity partially rescues dwarfism phenotype in CKO embryos in vivo. **A** Diagram of strategy about intraperitoneal injection of PBS or 3-BrPA and corresponding possible offspring. **B** General inspection and skeletal preparation of E18.5 embryos delivered from pregnant mice injected with PBS or 3-BrPA. Scale bar: 1 mm. **C** Quantification of humerus length (left panel), tibia length (middle panel), and vertebrae height (right panel) according to skeletal preparation of E18.5 embryos delivered from pregnant mice injected with PBS or 3-BrPA. Statistical analysis was exerted between CKO + PBS and CKO + 3-BrPA groups. *n* = 3 biological replicates. **D** H&E staining of tibia of E18.5 embryos delivered from pregnant mice injected with PBS or 3-BrPA. Boxes: magnification of resting zone in growth plate. Dotted circles: the area where atypical chondrocytes exist. Scale bar: 100 μm. **E** Immunostaining of BrdU assay, TUNEL assay, Sox9, Col10a1, and Mmp13 of tibia of E18.5 embryos delivered from pregnant mice injected with PBS or 3-BrPA and corresponding quantitative analysis. Statistical analysis was exerted upon CKO + PBS and CKO + 3-BrPA groups. Dotted lines: boundary between hypertrophic zone and POC. Dotted circles: the area where atypical chondrocytes exist. White arrows: positive range of Col10a1. *n* = 3 biological replicates. Scale bar: 100 μm. **F** von Kassa staining of tibia of E18.5 embryos delivered from pregnant mice injected with PBS or 3-BrPA and quantitative analysis. *n* = 3 biological replicates. Scale bar: 100 μm. *p*-value was calculated by one-way ANOVA followed by Tukey’s multiple comparisons tests. Data were shown as mean ± SD. *p < 0.05; **p < 0.01; ***p < 0.001; ns: not significant.

To test whether the excessive intensity of glycolysis detected in CKO embryos resulted in the dwarfism, we continuously injected pregnant mice with low-dosage 3-BrPA (7.5 mg/kg/d) [15, 39, 40] intraperitoneally daily (Fig. 6A). Since the CKO mice injected with 3-BrPA still died soon after delivery at P0 as the CKO mice injected with PBS, we sacrificed the pregnant mice at E18.5 to observe the rescue. First, we found that there was no difference between WT + PBS embryos and WT + 3-BrPA embryos (Fig. 6B, C), proving that low-dosage 3-BrPA had no influence on normal embryos. Then, we found that the height and limb length of CKO + 3-BrPA embryos were significantly larger than CKO + PBS embryos but still smaller than WT + PBS embryos or WT + 3-BrPA embryos (Fig. 6B, C). The range where atypical chondrocytes existed also significantly shrank in the growth plate of CKO + 3-BrPA embryos compared to CKO + PBS embryos (Fig. 6D). Compared to CKO + PBS embryos, CKO + 3-BrPA embryos exhibited increased proliferation and decreased apoptosis, and the number of Sox9 positive cells was elevated while the expression of Col10a1 and Mmp13 were also significantly decreased (Fig. 6E). Overall, these results indicated that suppressing excessive intensity of glycolysis partially rescued the dwarfism resulting from the loss of Hnrnpk in vivo.

## Discussion

The concordant proliferation and differentiation of growth plate chondrocytes are vital for endochondral ossification and the formation of long bones. Developmental abnormality of the growth plate mainly results in skeletal deformity including dwarfism, osteogenesis imperfecta, and osteoporosis [41]. In this research, for the first time, we discovered the essential role of Hnrnpk in maintaining the survival and function of chondrocytes during growth plate development, and the ablation of Hnrnpk resulted in the occurrence of dwarfism. Hnrnpk null growth plate chondrocytes exhibited damaged survival and premature differentiation. Furthermore, ablating Hnrnpk in hypertrophic chondrocytes led to enhanced transdifferentiation and increased bone mass. In terms of mechanism, Hnrnpk mediated the degradation of *Hif1a* mRNA to avoid the excessive activation of the Hif-1 signaling pathway under hypoxic condition and consequently guaranteed the appropriate intensity of glycolysis of chondrocytes. Collectively, our work highlights the regulatory role of Hnrnpk in the Hif1α-glycolysis axis during growth plate development.

In the Hnrnpk null growth plate, many disproportionately swollen chondrocytes called atypical chondrocytes were detected in the center of growth plate. Similar to our study, atypical chondrocytes have also been reported in mice lacking the von Hippel-Lindau tumor suppressor protein (pVHL) [42, 43], which was the E3 ubiquitin ligase regulating the process of proteasome degradation of Hif1α, and loss of pVHL resulted in the accumulation of Hif1α [44]. This research supported the finding that the presence of atypical chondrocytes in Hnrnpk null mice resulted from the increased expression of Hif1α. We also discovered that the atypical chondrocytes were Sox9 negative. Sox9 was an essential transcription factor maintaining the resting statues of the chondrocytes, and its downregulation promoted premature differentiation [1]. Thus, we deduced that atypical chondrocytes were one kind of premature differentiating chondrocytes.

We noticed that ablating Hnrnpk also led to osteosclerosis in POC because of the enhanced transdifferentiation from hypertrophic chondrocytes to osteoblasts. Though the osteoblasts in POC mainly originated from perichondrial osteoprogenitors [45], during the embryonic stage, about 30% of the osteoblasts originated from the hypertrophic chondrocytes of the growth plate [28, 46]. Previous research showed that the canonical Wnt signaling pathway, Iroquois homeobox-containing transcription factors IRX3, and IRX5 regulated the transdifferentiation of the hypertrophic chondrocytes, and their ablation resulted in the change of bone mass [47, 48]. However, the mechanism controlling the balance between apoptosis and transdifferentiation is not fully understood. In our study, we proved that ablating Hnrnpk contributed to the transdifferentiation of hypertrophic chondrocytes, but the osteosclerosis persisted after the phenotype was significantly rescued via suppressing excessive glycolysis (Fig. 6F), suggesting the change might not result from the Hif-1 signaling pathway. Even if the overexpression of Hif1α using *Prx1-Cre; Vhl*^*fl/fl*^ also led to the excessive trabecularization in POC, it mainly influenced the osteoblasts rather than chondrocytes [42]. Thus, the reason for the enhanced transdifferentiation in our data requires further study.

Hnrnpk is one kind of RNA binding protein, possessing the capacity of binding RNA and playing multiple roles in influencing the metabolism of RNA. In our research, for the first time, we illustrated that Hnrnpk bound to *Hif1a* mRNA and its ablation resulted in the increased half-life of *Hif1a* mRNA. Interestingly, under normoxic condition, the loss of Hnrnpk by *Ad-Cre* infection resulted in the increased expression of Pfkfb3 but not Ldha (Fig. 5D). Then, we also detected the significant binding between Hnrnpk and *Pfkfb3* mRNA (Supplementary Fig. S4A, B) and the increased half-life of *Pfkfb3* mRNA in Hnrnpk ablation chondrocytes (Supplementary Fig. S4C), suggesting that the increased expression of Pfkfb3 was partially independent of Hif1α. However, the mechanism that Hnrnpk degraded mRNA was still unknown. Previous studies proved that hnRNPK regulated the stability of mRNA via interacting with other proteins [25, 49, 50]. Because of the multifunction of hnRNPK, we hypothesized that Hnrnpk might interact with some other proteins to mediate the degradation of *Hif1a* mRNA, which was worthy of further study.

hnRNPK was proven to interact with long non-coding RNA *Lncenc1* to regulate the expression of glycolytic genes in mouse embryonic stem cells (ESCs) through the transcriptional mechanism and knocking out hnRNPK decreased the transcription of glycolytic genes [51]. In chondrocytes, we proved that Hnrnpk bound with the *Hif1a* mRNA and influenced the activity of the Hif-1 signaling pathway, then regulated the expression of glycolytic genes indirectly, and knocking out Hnrnpk elevated the intensity of the glycolysis. Glycolysis maintained the self-renewal of ESCs but helped to adapt the hypoxic condition in the growth plate chondrocytes. Thus, the intensity of the glycolysis in the chondrocytes relied more on the Hif-1 signaling pathway than in ESCs. Though we proved that Hnrnpk interacted indirectly with glycolytic genes in our study, Hnrnpk might also function as a transcription factor to regulate the expression of glycolytic genes through a transcriptional mechanism.

**Fig. 7:**
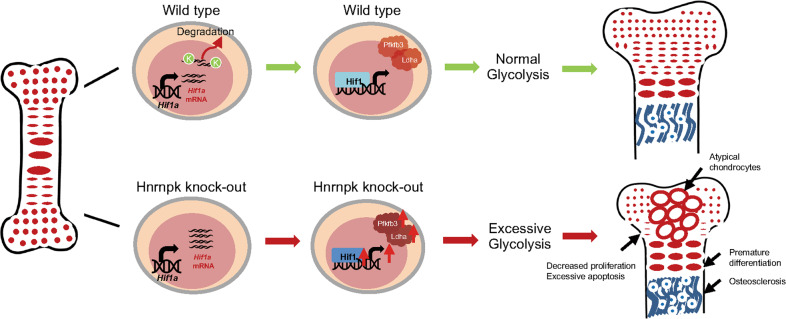
Diagram of Hnrnpk orchestrating growth plate development. During growth plate development, Hnrnpk bound to the *Hif1a* mRNA and mediated its degradation. Ablating Hnrnpk in chondrocytes resulted in the increased expression of Hif1α and then the increased expression of downstream target genes including Ldha and Pfkfb3. The elevated level of glycolytic enzymes led to excessive glycolysis and finally abnormal growth plate development.

In conclusion (Fig. 7), our present study elucidated that Hnrnpk was vital for concordant growth plate development by maintaining the appropriate intensity of glycolysis, and its ablation resulted in the occurrence of dwarfism. Our data supported the idea that Hnrnpk might act as an important regulator for adjusting glycolysis in chondrocytes, and the results offered a new insight into the therapeutic potential of Hnrnpk in skeletal deformity or osteoarthritis.

## Supplementary information

Reproducibility checklist

Supplementary material

Supplementary table

Supplementary material Western blots

## Data Availability

The RNA-seq data from this publication have been uploaded to the GEO database and assigned the identifiers GSE197283.
