# Supplementary material for: Hnrnpk maintains chondrocytes survival and function during growth plate development via regulating Hif1α-glycolysis axis
Source: Cell Death Dis. 2022 Sep 20;13(9):803. doi: 10.1038/s41419-022-05239-0 (PMC9489716; doi:10.1038/s41419-022-05239-0)
Supplement: Supplementary file 2 — Supplementary material [file 41419_2022_5239_MOESM2_ESM.docx]

**
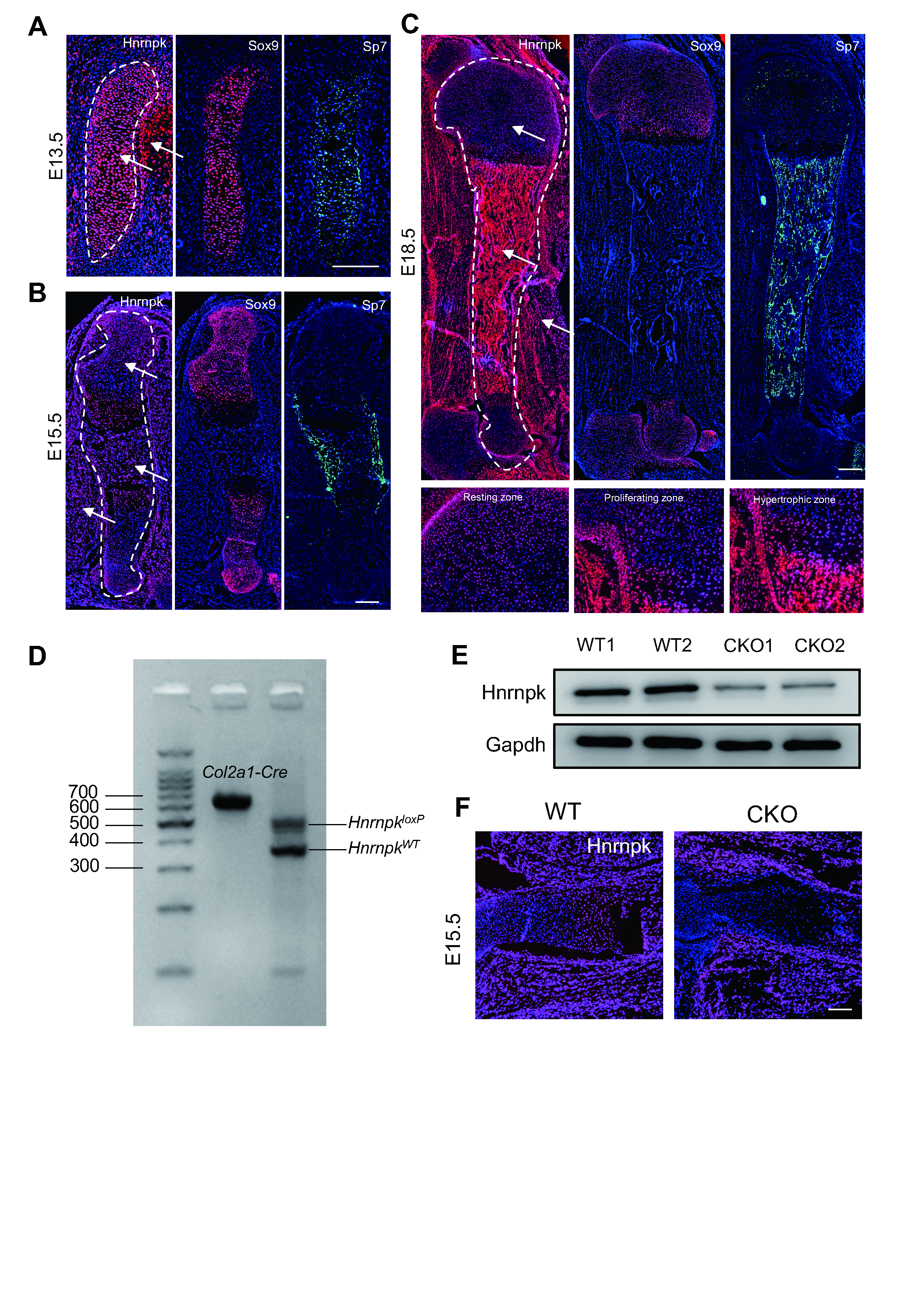
**

**Supplementary Fig. S1 Construction of *Hnrnpk* conditional knock-out mice.**

(A-C) Immunostaining of Hnrnpk, Sox9, and Sp7 in consecutive sections of the wild type humerus of E13.5 (A), E15.5 (B), and E18.5 (C) embryos. Dotted lines: humerus. Arrows: the expression of Hnrnpk in growth plate cartilage, bone marrow, and adjacent soft tissue. Scale bar: 100 μm.

(D) Gel image of genotype of *Col2a1-Cre* and *Hnrnpk^loxP^*.

(E) Hnrnpk protein level of the growth plate cartilage of E15.5 WT and CKO embryos.

(F) Immunostaining of Hnrnpk of the humerus of E15.5 WT and CKO embryos. Scale bar: 100 μm.

**
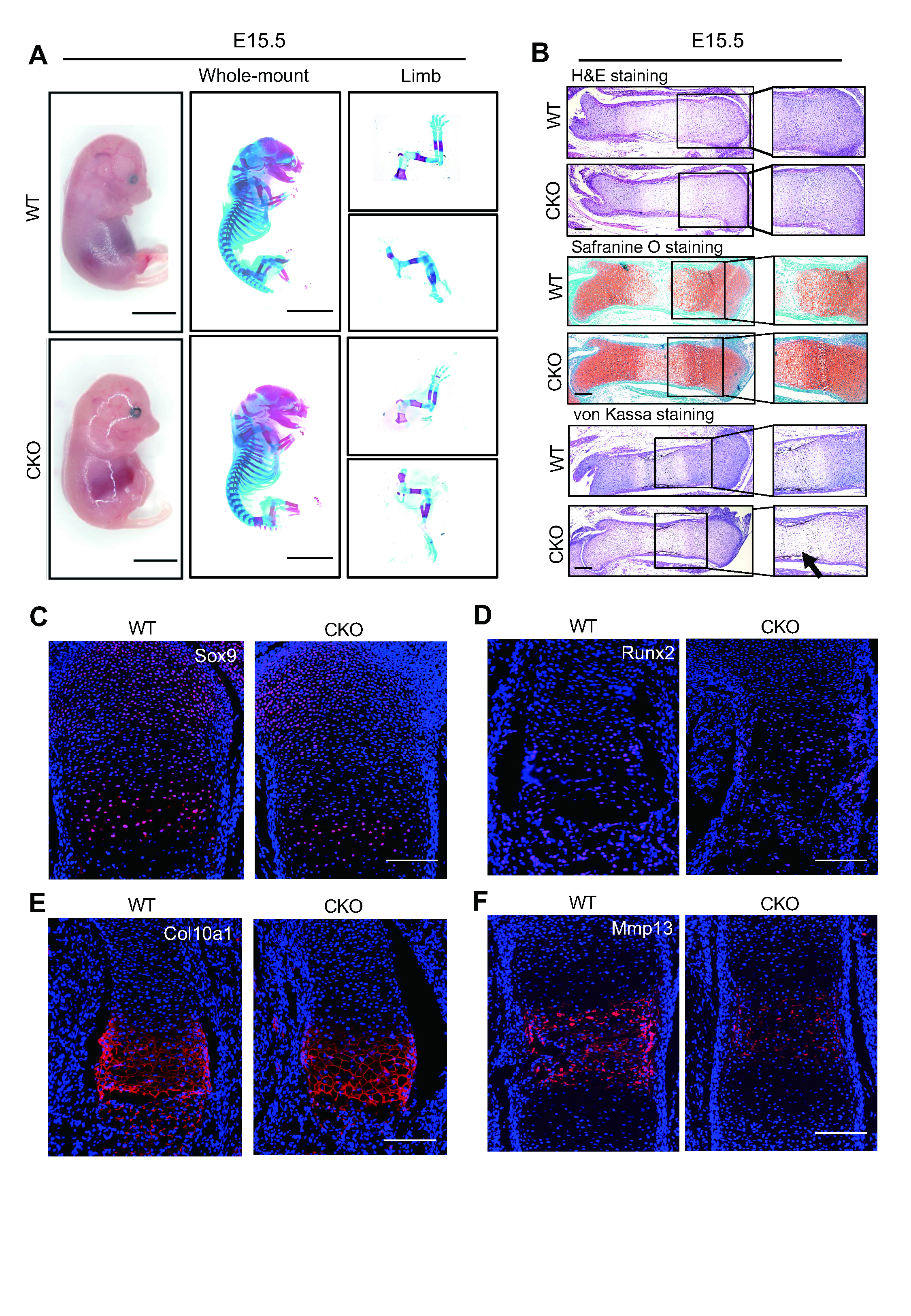
**

**Supplementary Fig. S2 Ablation of Hnrnpk in the chondrocytes results in delayed formation of** **primary ossification center of E15.5 CKO embryos.**

(A) General inspection (left panel) and skeletal preparation (middle and right panel) of E15.5 WT and CKO embryos. Scale bar: 500 μm.

(B) H&E staining (top), Safranine O staining (middle), and von Kassa staining (bottom) of femur of E15.5 WT and CKO. Black arrow: delay formation of POC. Scale bar: 100 μm.

(C-F) Immunostaining of Sox9 (C), Runx2 (D), Col10a1 (E), and Mmp13 (F) of tibia of E15.5 WT and CKO. Scale bar: 100 μm.

**
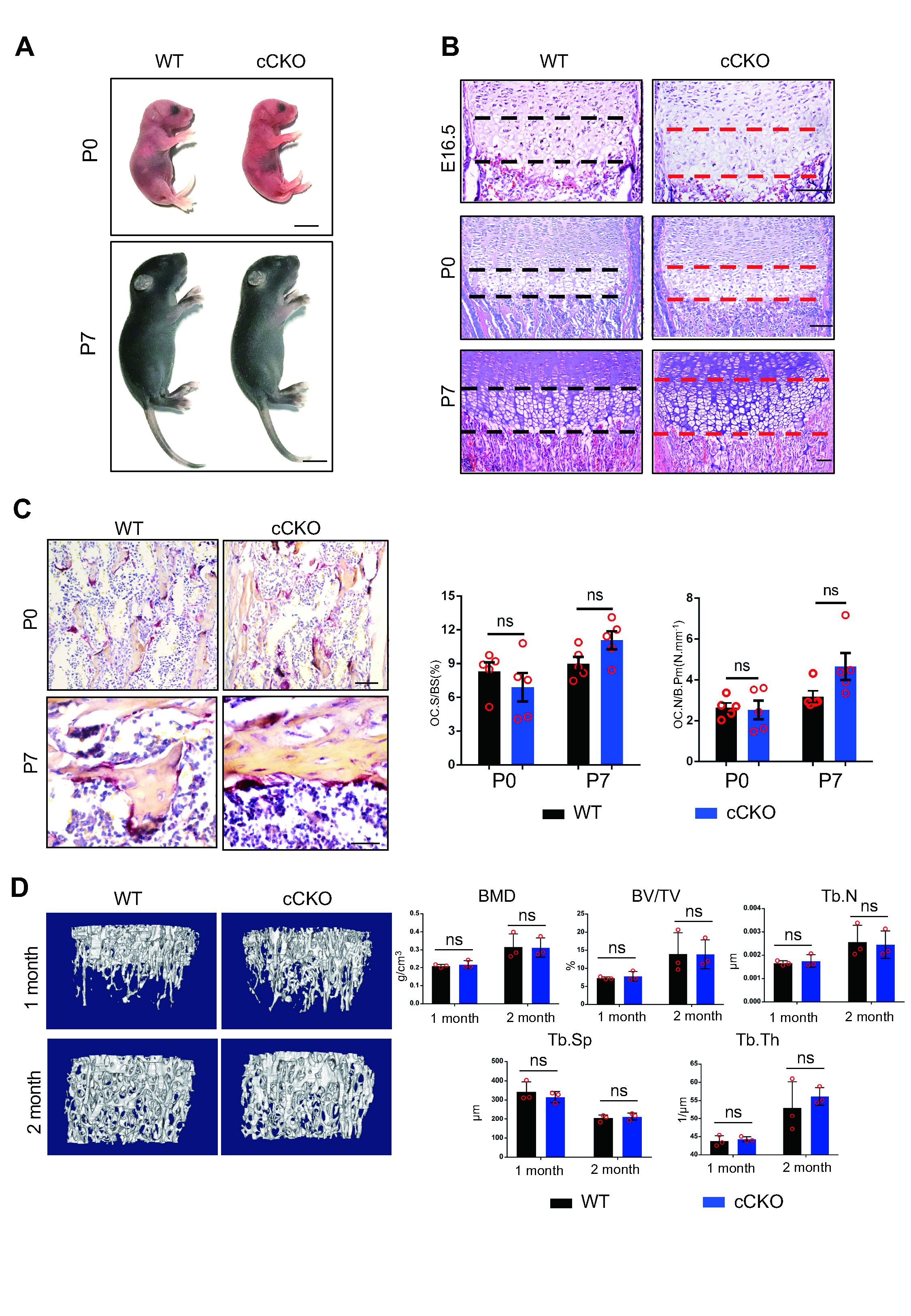
**

**Supplementary Fig. S3 Loss of Hnrnpk in hypertrophic chondrocytes increases bone mass.**

(A) General inspection of P0 (top) and P7 (bottom) WT and cCKO mice. Scale bar: 1 mm.

(B) H&E staining of tibia of E16.5 (left panel), P0 (middle panel), and P7 (right panel) WT and cCKO mice. Dotted lines: range of hypertrophic chondrocytes. Scale bar: 100 μm.

(C) TRAP staining in POC of tibia of P0 (top) and P7 (bottom) WT and cCKO mice and the quantification of OC.S/BS (area of osteoclast surface/area of bone surface) and OC.N/B.Pm (number of osteoclast/perimeter of bone surface). *n* = 5 biological replicates. Scale bar: 100 μm.

(D) Micro-CT scanning of femur of one-month and two-month old WT and cCKO mice and quantification of BMD, BV/TV, Tb.N, Tb.Sp, and Tb.Th. *n* = 3 biological replicates.

*p*-value was calculated by two-tailed unpaired Student’s t-test. Data was shown as mean ± SD. ns: not significant.

**
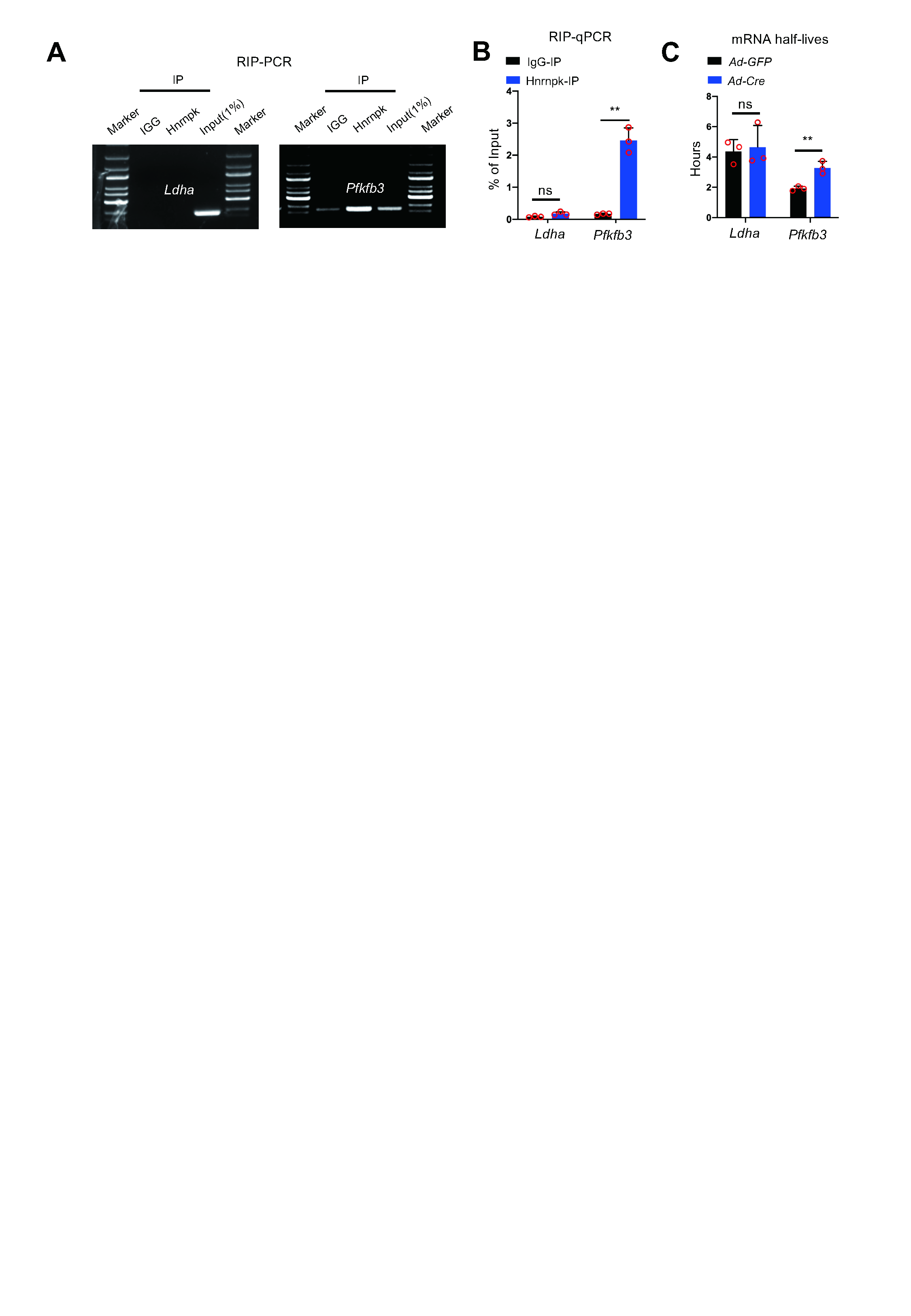
**

**Supplementary Fig. S4 Hnrnpk partially influences the stability of *Pfkfb3* mRNA.**

(A-B) RIP-PCR (A) and RIP-qPCR (B) were exerted to determine the level of binding between Hnrnpk and *Ldha* mRNA (left panel) or *Pfkfb3* mRNA (right panel). *n* = 3 biological replicates. IgG IP was used as specificity control.

(C) mRNA half-lives of *Ldha* and *Pfkfb3*. *n* = 3 biological replicates.

*p*-value was calculated by two-tailed unpaired Student’s t-test. Data was shown as mean ± SD. ***p* < 0.01; ns: not significant.
