# Supplementary material for: Hnrnpk maintains chondrocytes survival and function during growth plate development via regulating Hif1α-glycolysis axis
Source: Cell Death Dis. 2022 Sep 20;13(9):803. doi: 10.1038/s41419-022-05239-0 (PMC9489716; doi:10.1038/s41419-022-05239-0)
Supplement: Supplementary file 3 — Supplementary table [file 41419_2022_5239_MOESM3_ESM.docx]

**Supplementary Table 1.** Primers sequences for qPCR.

|  | | |
| --- | --- | --- |
| Gene | Direction | Sequences (5'-3') |
| *Actb* | forward | GGCTGTATTCCCCTCCATCG |
|  | reversed | CCAGTTGGTAACAATGCCATGT |
| *Hnrnpk* | forward | CCGTACAGACTACAATGCCAG |
|  | reversed | GCCCTCTTCCAAGGTAGGGAT |
| *Sox9* | forward | AGTACCCGCATCTGCACAAC |
|  | reversed | ACGAAGGGTCTCTTCTCGCT |
| *Col10a1* | forward | TTCTGCTGCTAATGTTCTTGACC |
|  | reversed | GGGATGAAGTATTGTGTCTTGGG |
| *Runx2* | forward | GACTGTGGTTACCGTCATGGC |
|  | reversed | ACTTGGTTTTTCATAACAGCGGA |
| *Mmp13* | forward | TGTTTGCAGAGCACTACTTGAA |
|  | reversed | CAGTCACCTCTAAGCCAAAGAAA |
| *Bax* | forward | TGAAGACAGGGGCCTTTTTG |
|  | reversed | AATTCGCCGGAGACACTCG |
| *Cdkn1a* | forward | CCTGGTGATGTCCGACCTG |
|  | reversed | CCATGAGCGCATCGCAATC |
| *Pcna* | forward | TTTGAGGCACGCCTGATCC |
|  | reversed | GGAGACGTGAGACGAGTCCAT |
| *Matn1* | forward | GACCTGGTGTTTGTTGTCGAT |
|  | reversed | TGAGACAGGAATACCTTCACCTT |
| *Comp* | forward | ACTGCCTGCGTTCTAGTGC |
|  | reversed | CGCCGCATTAGTCTCCTGAA |
| *Pycr1* | forward | ATGAGCGTAGGCTTCATCGG |
|  | reversed | GTGTCAGGTTCACCCCTATCT |
| *Angptl6* | forward | TTGGGCGTCCAGAAGGAGAA |
|  | reversed | CAGTCCTCTAGGAGTATCAGCAG |
| *Cnmd* | forward | TAGGGCCTGAGGACGTTGAG |
|  | reversed | CAGCTCCTACCTTGAGCAGC |
| *Ucma* | forward | TTTCATGCAAGAATCTGATGCCT |
|  | reversed | ACTTCATCTCGGGACTTAGGAG |
| *Hif1α* | forward | TCTCGGCGAAGCAAAGAGTC |
|  | reversed | AGCCATCTAGGGCTTTCAGATAA |
| *Glut1* | forward | GCAGTTCGGCTATAACACTGG |
|  | reversed | GCGGTGGTTCCATGTTTGATTG |
| *Pgk1* | forward | ATGTCGCTTTCCAACAAGCTG |
|  | reversed | GCTCCATTGTCCAAGCAGAAT |
| *Ldha* | forward | TGGAAGACAAACTCAAGGGCGAGA |
|  | reversed | TGACCAGCTTGGAGTTCGCAGTTA |
| *Pfkfb2* | forward | GACTGCAACAGCAGCTATAAAC |
|  | reversed | GCTGGCAAGCCAATCATAAC |
| *Pfkfb3* | forward | CTACCTCAACTGGATAGGTGTTC |
|  | reversed | AGGGCGGAAGAAGTTGTAAG |
| *Pfkfb4* | forward | CATCCAGAGTCGCATCGTTTA |
|  | reversed | CTTGAGGTTTAGCTCGCTCTC |
| *Eno2* | forward | AGGTGGATCTCTATACTGCCAAA |
|  | reversed | GTCCCCATCCCTTAGTTCCAG |
| *Eno3* | forward | CACAGCCAAGGGTCGATTCC |
|  | reversed | AGGTATCGTGCTTTGTCTCCA |
| *Ndufc1* | forward | CATCTTCATTGTGTGTTTGGATGA |
|  | reversed | TCAACACGGTCGAAGTTCTATG |
| *Ndufaf2* | forward | ACTGGAGAGGGCAGACTATT |
|  | reversed | GGTGGAGTCTTCCTTGTTCTTC |
| *Ndufaf4* | forward | CCCAGCTTGATTCCTCTCTTC |
|  | reversed | GGCTCTGGGTTGTTCTTTCT |
| *Nfatc1* | forward | GAGACAGACATCGGGAGGAAGA |
|  | reversed | GTGGGATGTGAACTCGGAAGA |
