# Supplementary figures and images for: Hnrnpk maintains chondrocytes survival and function during growth plate development via regulating Hif1α-glycolysis axis
Source: Cell Death Dis. 2022 Sep 20;13(9):803. doi: 10.1038/s41419-022-05239-0 (PMC9489716; doi:10.1038/s41419-022-05239-0)

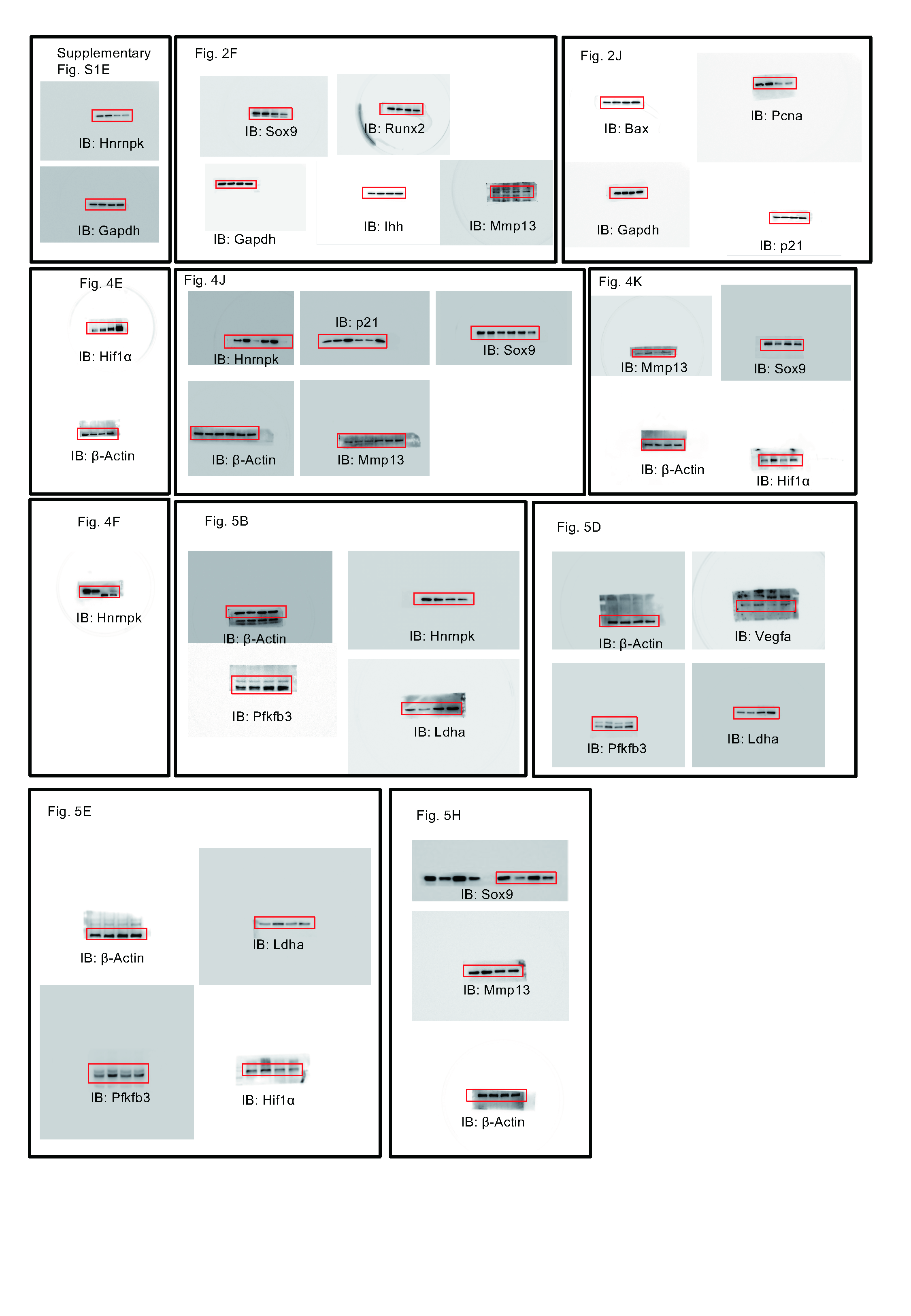


**Supplementary material Western blots.** Original files of Western blots.

Supplement: Supplementary file 4 — Supplementary material Western blots [file 41419_2022_5239_MOESM4_ESM.docx]
